# Evaluation of Paraspinal Muscle Epimuscular Fat in Subjects With Low Back Pain in a Tertiary Care Setting

**DOI:** 10.7759/cureus.84714

**Published:** 2025-05-23

**Authors:** Thavan Mummaneni, Harini Bopaiah, Anil K Sakalecha, Guru Yogendra Muthyal, Vamsi Venkat

**Affiliations:** 1 Department of Radiodiagnosis, Sri Devaraj Urs Medical College, Kolar, IND

**Keywords:** epimuscular fat (ef), erector spinae (es), low back pain (lbp), magnetic resonance imaging (mri), multifidus (mf), paraspinal muscles

## Abstract

Introduction: Low back pain (LBP) is among the most commonly encountered health complaints globally, significantly affecting the person's quality of life and imposing a substantial financial burden on individuals and society. Structural alterations in paraspinal muscles, particularly fatty infiltration, have been associated with chronic LBP. However, limited studies have specifically focused on the role of epimuscular fat (EF) in LBP, and it remains less understood.

Objective: This study aimed to evaluate the distribution of paraspinal muscle EF in patients with chronic nonspecific LBP and to investigate its associations with demographic parameters, body mass index (BMI), and the severity of LBP.

Materials and methods: A cross-sectional study was conducted involving 56 participants presenting with chronic nonspecific LBP at RL Jalappa Hospital and Research Centre, a rural tertiary care hospital attached to Sri Devaraj Urs Medical College in Tamaka, Kolar, Karnataka, India. EF was graded on a 0-5 qualitative scale for multifidus (MF) and erector spinae (ES) paraspinal muscles at lumbar intervertebral disc levels on T2 axial magnetic resonance imaging (MRI) sequences acquired using Siemens® MAGNETOM Avanto (Siemens Healthineers, Erlangen, Germany), a Tim+Dot 1.5 Tesla, 18-channel MRI machine. The Oswestry Disability Index (ODI) questionnaire was used for LBP severity assessment. Demographic data, including age, sex, and BMI, were recorded. Spearman's correlation and comparative analyses using independent t-tests, multiple group comparison tests, and multiple linear regression were performed to evaluate associations between EF and clinical-demographic variables.

Results: Fifty-six participants (mean age 45.3±12.7 years; BMI 27.4±4.5 kg/m²; 46.4% female) were included. EF scores increased from L1-L2 (2.1±0.8) to L4-L5 (7.2±1.4) and L5-S1 (6.8±1.2). EF showed significant positive correlations with age (r=0.45) and BMI (r=0.52) (both p<0.001). Women had higher EF scores than men (p=0.017). EF also increased across BMI categories (p=0.033) and ODI-based LBP severity levels (p=0.0036). Regression analysis identified age, BMI, sex, and ODI severity as independent EF predictors (R²=0.44; adjusted R²=0.41).

Conclusion: EF accumulation correlates significantly with age, BMI, sex, and chronic LBP severity, with a predilection for lower lumbar levels suggestive of biomechanical adaptation. These findings highlight EF as a potential biomarker and therapeutic target. Interventions aimed at EF reduction may enhance spinal stability and alleviate symptoms. Further longitudinal studies are warranted to validate these associations.

## Introduction

Low back pain (LBP) is among the most common musculoskeletal complaints, globally affecting individuals belonging to various age groups and socioeconomic backgrounds. Research indicates that about 80% of people will suffer from LBP at some point, making it an important cause of disability that impacts the daily work life and productivity of people, with a significant financial burden on the economy [1]. Chronic LBP is defined as symptomatic pain for three months or more with a multifaceted etiology, complicating its management and need for integrated diagnostic and treatment approaches [2]. LBP arises from a combination of anatomical, functional, psychological, and environmental factors, highlighting the importance of understanding the anatomical and functional elements involved [3].

The paraspinal muscles, especially the multifidus (MF) and erector spinae (ES) muscles, are responsible for spinal alignment and mechanical stability, as they effectively distribute weights on the axial skeleton, facilitating functional movement. In individuals with chronic LBP, these paraspinal muscles experience structural changes like atrophy and fat replacement, which can compromise spinal stability [4]. Fat infiltration (FI) refers to fat accumulation within the muscle, replacing normal contractile tissue, whereas epimuscular fat (EF) is the deposition of fat externally between the muscle sheath (epimysium) and adjacent fascia, impacting muscle function structurally and functionally [5].

EF may influence spinal biomechanical properties by causing changes in the structural and functional integrity of the paraspinal muscle-tendon units. Such alterations could have a negative impact on efficient force transmission during movement, thereby compromising muscle performance [6]. While the association between intramuscular fat and muscle degeneration has been well documented in various literary sources, there is less literature on the role of EF in spinal disorders and LBP, indicating a significant research gap. EF is anatomically distinct from FI; its clinical implications with respect to LBP are still under investigation [7,8].

Magnetic resonance imaging (MRI) is considered the most precise imaging technique for evaluating muscle tissue due to its high resolution and ability to distinguish muscle from fat. It offers clear visualization and quantification of FI and EF [9]. Advanced techniques, such as the Dixon method, enhance the accuracy of fat content assessment and the detection of subtle changes in muscle structure. However, inconsistency in scanning parameters and image planning and segmentation across different studies has shown variations in reported outcomes, limiting cross-study comparisons and applicability [5,6].

Recent studies show that EF accumulates more frequently in the lower levels of the lumbar spine, which are typically exposed to higher mechanical stress and are more prone to degenerative changes [7,8]. This distribution pattern is either due to an adaptive response to chronic weight loading or due to a pathological factor contributing to functional impairment [10].

Furthermore, demographic variables such as age, sex, and body mass index (BMI) appear to influence EF levels. Increased age is associated with fat accumulation within muscle, potentially leading to functional decline [11], while higher BMI correlates with elevated fat deposition around muscles, possibly increasing LBP symptoms through structural and functional alterations in paraspinal muscles [12]. Despite the associations, the complex interaction between demographic factors and EF deposition in LBP needs further investigation.

A recent study by Rosenstein et al. identified a significantly higher prevalence of EF at the L4-L5 and L5-S1 levels in patients with LBP as compared to healthy individuals, with no significant changes observed in the upper lumbar regions. They also found positive correlations between total EF scores and factors such as age, BMI, sex, and LBP status [7].

Vitale et al. demonstrated that EF is most frequently observed at the L3 and L4 vertebral levels, followed by the L5 vertebral level. They also observed that the area of EF is significantly larger at the lower lumbar levels and revealed a good positive correlation with BMI. Furthermore, a moderate correlation between EF and intramuscular FI was identified. However, this correlation did not retain significance after adjustment. In the adjusted models, EF continued to demonstrate an independent association with LBP intensity [8].

Considering this growing evidence, the current study seeks to investigate the distribution and role of EF in individuals with chronic nonspecific LBP. Through standardized MRI assessment, the study aims to clarify the relationship between EF, LBP severity, and demographic factors, thereby expanding current understanding and potentially informing future clinical evaluation and management strategies.

## Materials and methods

Study design

This study was designed as a cross-sectional observational study to evaluate the distribution and impact of EF in patients with chronic LBP, to assess the associations of EF with various demographic and clinical factors. This design facilitated the examination of potential correlations between EF and variables such as age, sex, BMI, and LBP severity.

Study setting

The study was conducted at a radiology department in RL Jalappa Hospital and Research Centre, a rural tertiary care hospital attached to Sri Devaraj Urs Medical College in Tamaka, Kolar, Karnataka, India, equipped with Siemens® MAGNETOM Avanto (Siemens Healthineers, Erlangen, Germany), a Tim+Dot 1.5 Tesla, 18-channel MRI machine to acquire axial and sagittal sections of the lumbar spine, for the precise assessment of paraspinal muscle morphology and composition. The department serves a diverse patient population, encompassing individuals referred for chronic LBP evaluation and management, thereby providing an appropriate setting for recruiting study participants.

Study duration

The study was done in three months, from January 1, 2025, to March 31, 2025. This timeframe encompassed all phases of the research, including participant recruitment, data collection, imaging procedures, data analysis, and preparation of findings for dissemination. The three-month duration was deemed sufficient to enroll the required sample size and complete all necessary assessments within the constraints of the study.

Participants

Inclusion Criteria

Individuals aged 18-60 years and individuals complaining of nonspecific LBP persisting for three months or longer were included in the study.

Exclusion Criteria

Participants were excluded if they had a history of spinal surgery or vertebral fractures; spinal deformities such as scoliosis; comorbid conditions limiting participation in imaging or assessments; or contraindications to MRI, including claustrophobia or metallic implants. Additionally, individuals with significant spinal pathologies identified on MRI (such as sacroiliitis, disc herniation, marked osteophytes, Baastrup disease, spinal canal stenosis, or spondylolisthesis) were excluded to minimize structural confounding.

Study sample

Fifty-six participants were selected using a prospective sampling method from the pool of patients presenting to the Department of Radiodiagnosis with chronic nonspecific LBP. Consecutive sampling was employed to minimize selection bias, wherein every eligible individual who met the inclusion criteria was included during the study period until a sufficient sample size was obtained.

Study sample size calculation

A total sample size was determined based on the correlation coefficient of FI percentage with the total qualitative EF score, as reported by Vitale et al. [8]. Utilizing a correlation coefficient (r) of 0.37, an alpha (α) of 0.05, and a power of 80%, which yielded a required sample size of 56 participants. The formula used for estimating the sample size was as follows: $n = \frac{(Z_{\alpha/2} + Z_{\beta})^2}{\left(0.5 \times \ln\left(\frac{1 + r}{1 - r}\right)\right)^2}$.

Study groups

As the study was cross-sectional and aimed at evaluating the distribution and associations of EF within a single population of patients with chronic LBP, no distinct study groups were formed. All participants constituted a single cohort, and comparisons were made within this group based on demographic and clinical variables such as age, sex, BMI, and severity of LBP.

Study procedure, parameters, and data collection

After obtaining ethical approval, eligible participants were recruited and provided detailed study information. Following informed consent, MRI was performed using a Siemens® MAGNETOM Avanto, Tim+Dot 1.5 Tesla, 18-channel system. Axial and sagittal T1- and T2-weighted sequences were acquired over the lumbar spine with a slice thickness of 4 mm. To enhance visualization and delineation of EF, axial fat-suppressed T2-weighted images were also included. Imaging data were stored securely, and all images were reviewed by trained radiologists blinded to the clinical details of participants to ensure objectivity in EF scoring.

**Figure 1:**
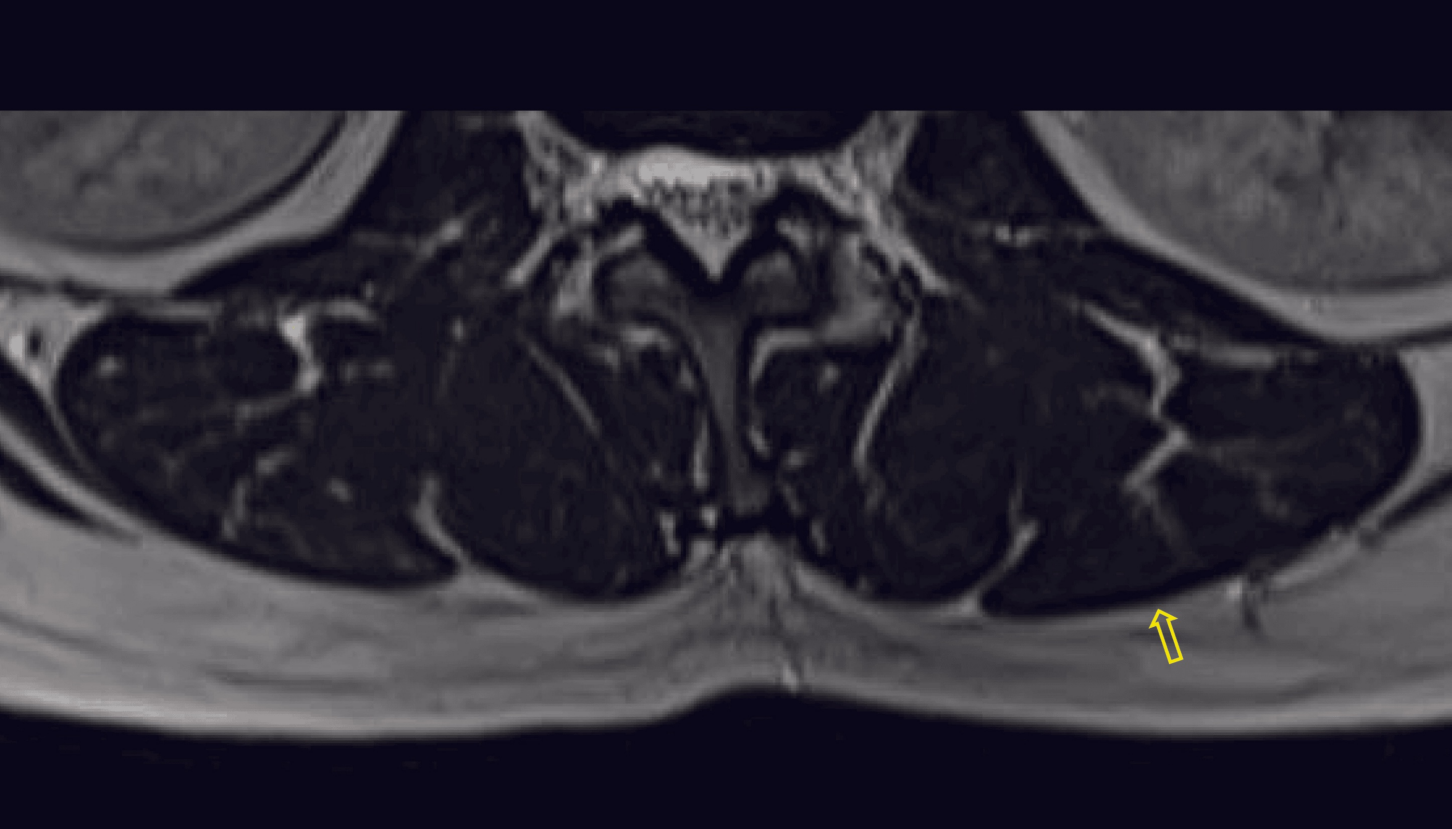
Axial T2-weighted MR image with yellow arrow along the posterior border of paraspinal muscles showing no epimuscular fat deposition, with a score of 0 MR: magnetic resonance

**Figure 2:**
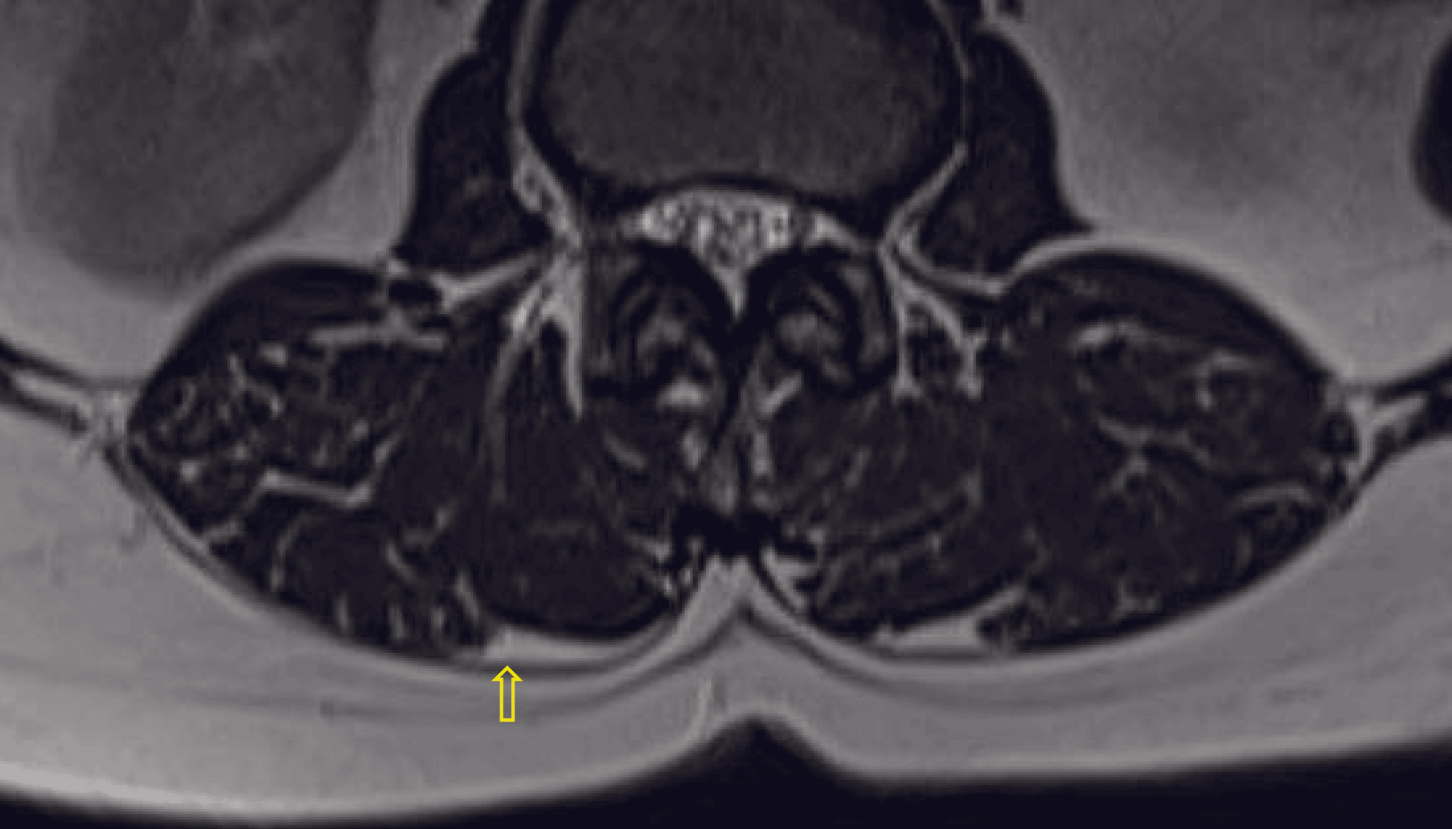
Axial T2-weighted MR image with yellow arrow pointing the epimuscular fat deposition along the posterior border of paraspinal muscles, with a score of 1 MR: magnetic resonance

**Figure 3:**
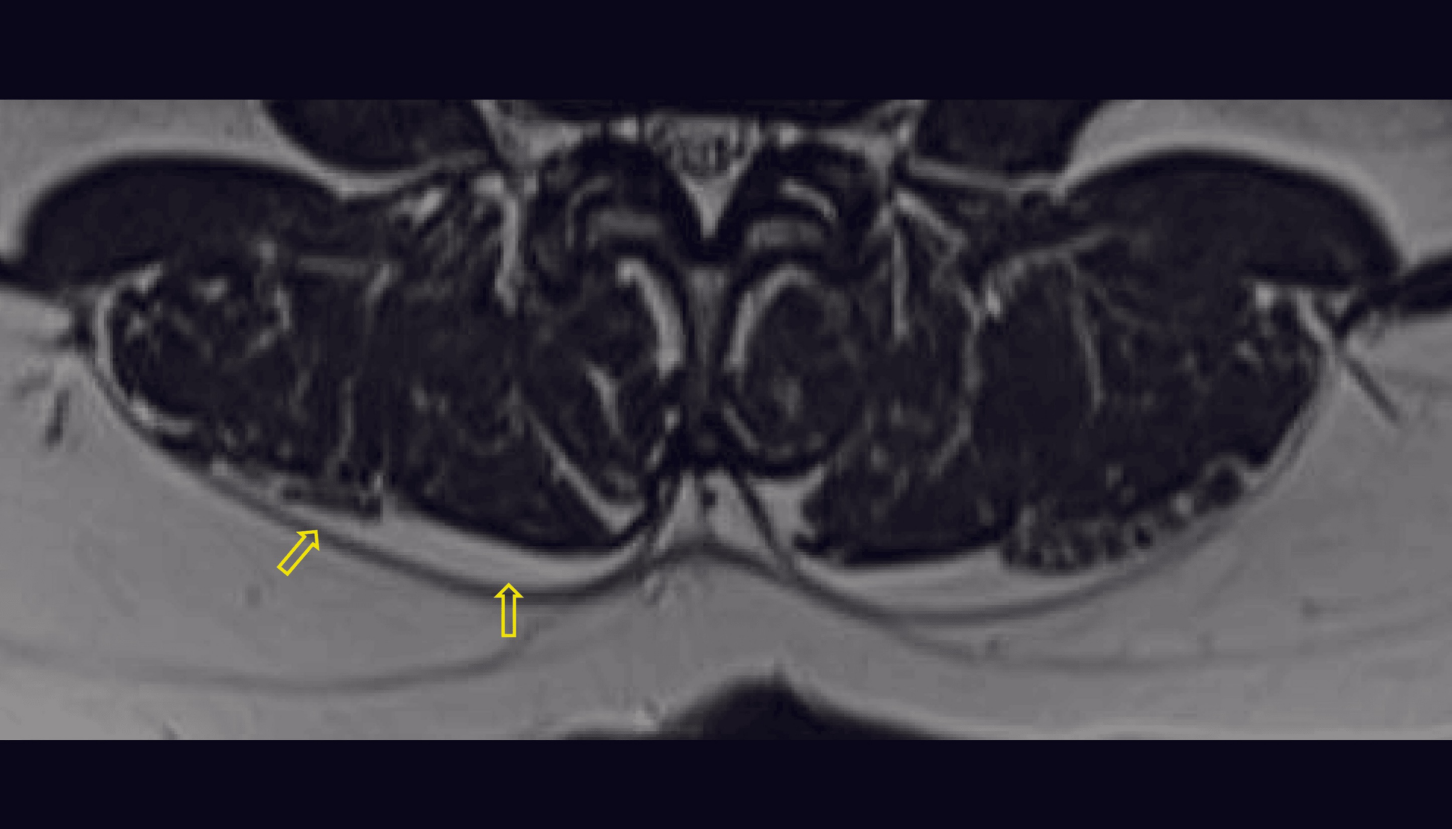
Axial T2-weighted MR image with yellow arrows pointing the epimuscular fat deposition along the posterior border of paraspinal muscles, with a score of 2 MR: magnetic resonance

**Figure 4:**
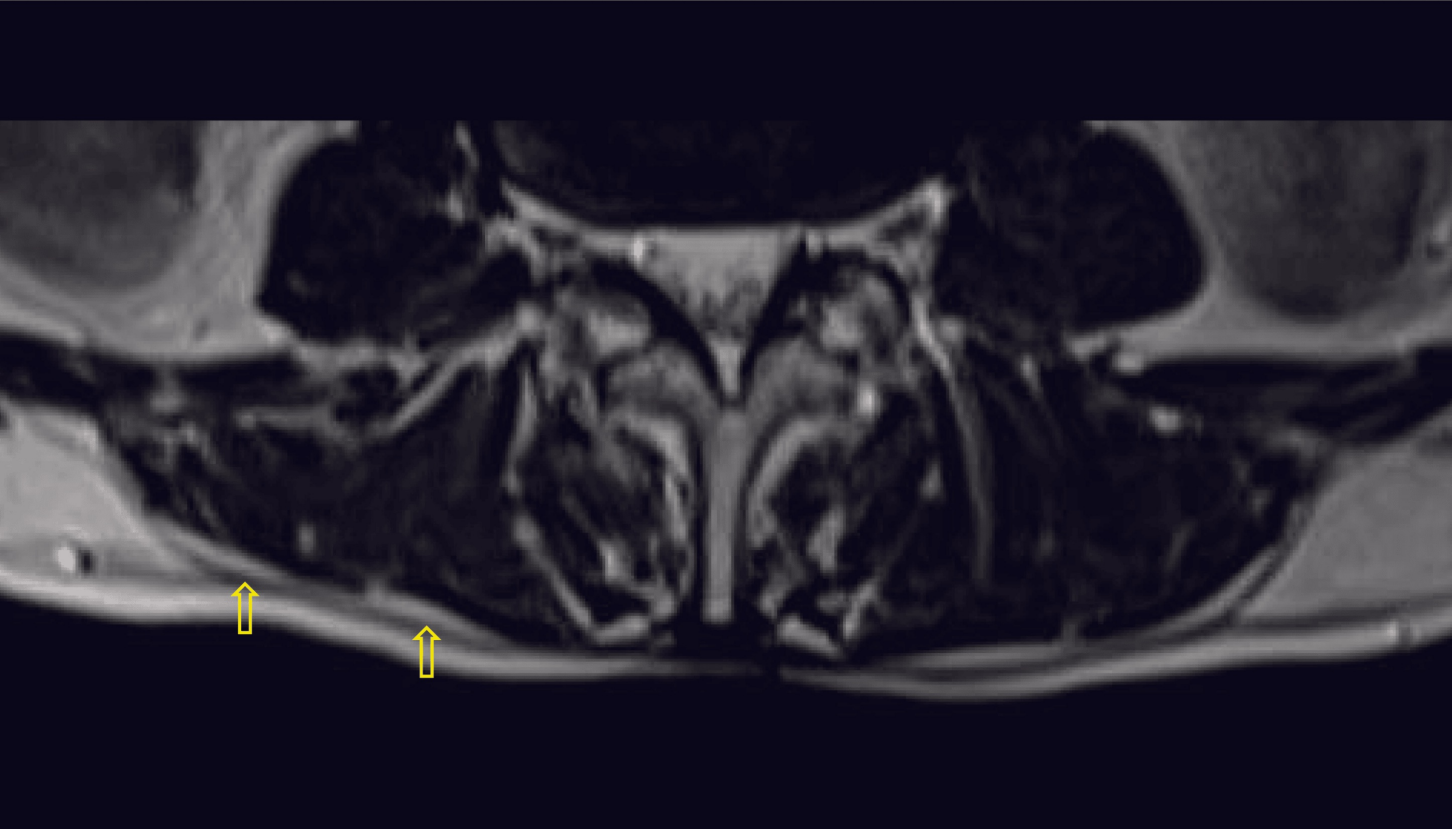
Axial T2-weighted MR image with yellow arrows pointing the epimuscular fat deposition along the posterior border of paraspinal muscles, with a score of 3 MR: magnetic resonance

**Figure 5:**
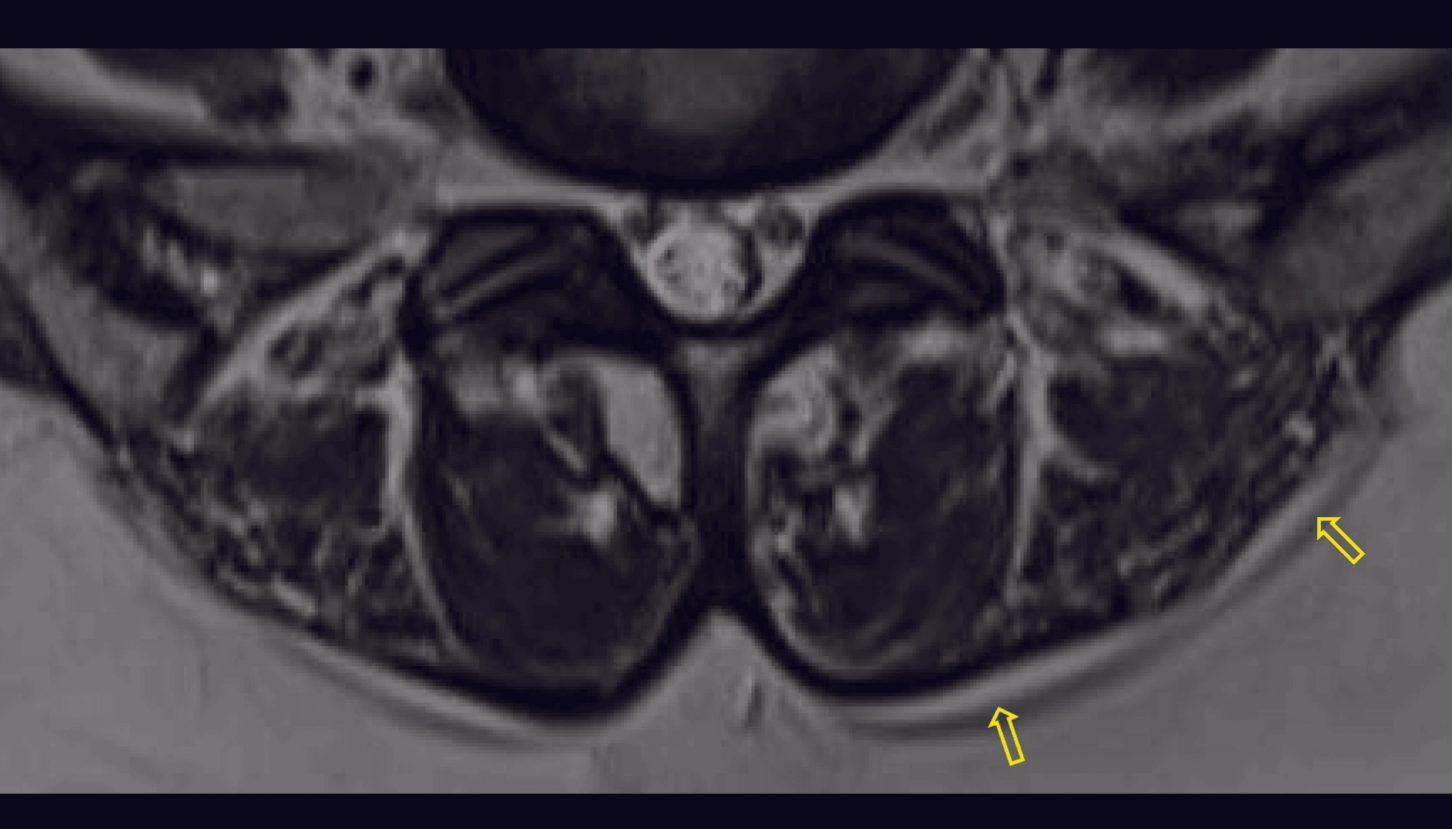
Axial T2-weighted MR image with yellow arrows pointing the epimuscular fat deposition along the posterior border of paraspinal muscles, with a score of 4 MR: magnetic resonance

**Figure 6:**
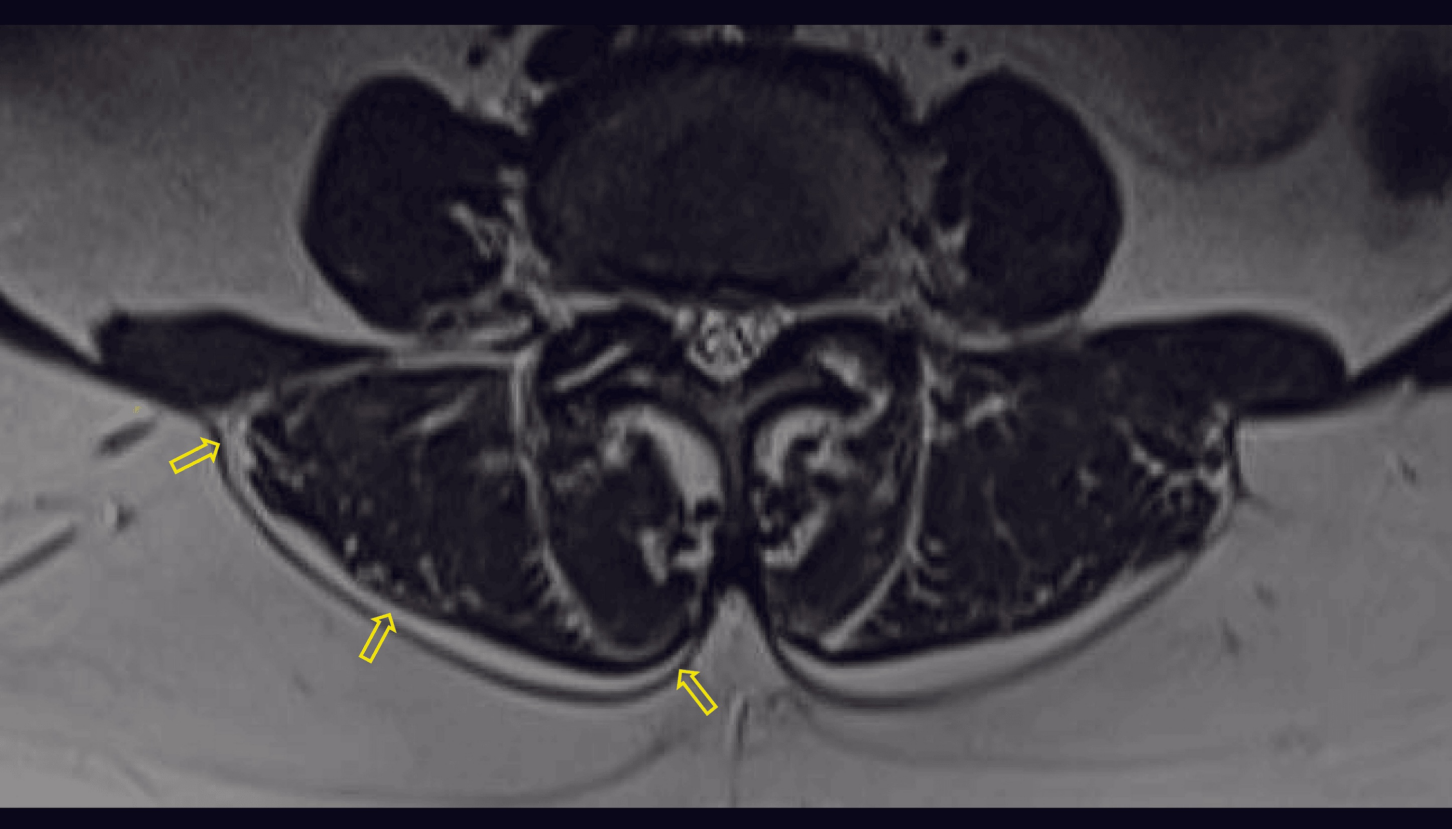
Axial T2-weighted MR image with yellow arrows pointing the epimuscular fat deposition along the posterior border of paraspinal muscles, with a score of 5 MR: magnetic resonance

EF scores were assessed using a 5-point qualitative scoring scale, originally described by Rosenstein et al. and detailed in Table 1 [7]. Representative MR images corresponding to each score are presented in Figures 1-6. In this grading system, a score of 0 indicates no visible EF along the posterior border of the paraspinal muscles, whereas a score of 5 reflects continuous EF accumulation along the entire posterior border of both the ES and MF muscles. For each participant, a total qualitative EF score was derived by summing the ratings across all lumbar intervertebral disc levels (L1-S1) on both the left and right sides. This composite score served as an index of overall paraspinal EF accumulation.

**Table 1:** EF scoring scale EF: epimuscular fat; ES: erector spinae; MF: multifidus Adapted with permission from Rosenstein et al. [7]

EF score rating	EF accumulation along the posterior border of the paraspinal muscle
Score 0	0% EF along paraspinal muscles
Score 1	1-25% EF along ES muscles
Score 2	25-50% EF along ES muscles
Score 3	51-75% EF along ES muscles
Score 4	76-100% EF along ES muscles
Score 5	100% EF along ES and MF muscles

Demographic and clinical data

Data were collected through structured questionnaires, including age, sex, height, weight (for BMI calculation), and duration of LBP. Additionally, participants completed the Oswestry Disability Index (ODI) questionnaire to assess the degree of pain-related functional impairment. The primary parameters assessed in this study included EF scores, LBP severity scores, and demographic factors (age, sex, and BMI), which were recorded to explore their associations with EF. All data were entered into a Microsoft Excel spreadsheet (Microsoft Corporation, Redmond, Washington, United States), ensuring accuracy and completeness. Confidentiality was maintained by assigning unique identification numbers to each participant, with no personally identifiable information included in the data analysis.

Data analysis was performed using IBM SPSS Statistics for Windows, Version 25.0 (Released 2017; IBM Corp., Armonk, New York, United States). The following steps were undertaken: For descriptive statistics, data were calculated as means and standard deviations or frequencies and proportions for continuous or categorical variables, wherever applicable. For correlation and comparative analysis, Spearman's correlation, independent t-tests, and multiple group comparison tests were used wherever required for statistical analysis while considering a p-value of <0.05 as statistically significant, indicating a meaningful association or difference. For data visualization, tabular representations were used to illustrate the distribution of EF across lumbar levels and its associations with demographic and clinical factors.

All statistical procedures adhered to the assumptions of the tests used, and appropriate measures were taken to address any violations, such as non-normal distribution of variables.

Ethical considerations

Prior to initiation, ethical clearance was secured from the Central Ethics Committee of Sri Devaraj Urs Academy of Higher Education and Research (approval number: SDUAHER/KLR/R&D/CEC/S/PG/129/2024-25; date: 11-02-2025).

## Results

A total of 56 participants were included in the study. The mean age of the cohort was 45.3 years (±12.7). In terms of sex distribution, men constituted 53.6% (n=30), while women were 46.4% (n=26) of the participants. The average BMI of total participants measures 27.4 kg/m² (±4.5), suggesting that most participants come under the overweight range. The mean duration of LBP reported by participants was 12.5 months (±6.8). Additionally, the average ODI was 33.2% (±25%), indicating a moderate degree of functional impairment due to LBP. These baseline characteristics are depicted in Table 2. 

**Table 2:** Study population baseline demographic and clinical data N: number of participants; SD: standard deviation; BMI: body mass index; LBP: low back pain; ODI: Oswestry Disability Index

Characteristic	N (%)	Mean±SD
Age (years)	-	45.3±12.7
Sex		
Male	30 (53.6%)	-
Female	26 (46.4%)	
BMI (kg/m²)	-	27.4±4.5
LBP duration (months)	-	12.5±6.8
ODI (%)	-	33.2±25

EF distribution was evaluated across the lumbar spine from L1-L2 to L5-S1. A clear increasing trend in mean EF scores was observed from the upper to the lower lumbar levels. The lowest EF accumulation was noted at L1-L2 (2.1±0.8), while the highest levels were recorded at L4-L5 (7.2±1.4) and L5-S1 (6.8±1.2), as depicted in Table 3. These findings indicate a greater predisposition for FI in the lower lumbar segments, likely attributed to their increased mechanical loading and critical role in maintaining spinal mobility and stability.

**Table 3:** Mean EF scores at each lumbar intervertebral disc level EF: epimuscular fat; SD: standard deviation

Lumbar level	Mean EF score (±SD)
L1-L2	2.1±0.8
L2-L3	3.2±1.0
L3-L4	5.0±1.2
L4-L5	7.2±1.4
L5-S1	6.8±1.2

A moderate positive correlation was observed between age and total EF scores (r=0.45; p<0.001), as depicted in Table 4. This indicates that EF accumulation increases with advancing age, which highlights that aging may play a contributory role in FI within the paraspinal muscles, potentially impairing muscular support and exacerbating susceptibility to LBP.

**Table 4:** Correlation between EF scores and age and EF scores and BMI EF: epimuscular fat; BMI: body mass index *: statistically significant

Variable pair	Correlation coefficient (r)	P-value
Age and total EF score	0.45	<0.001^*^
BMI and total EF score	0.52	<0.001^*^

Additionally, a strong positive correlation was found between BMI and total EF scores (r=0.52; p<0.001) as depicted in Table 4. This association implies that individuals with higher BMI tend to exhibit greater EF deposition, likely due to increased systemic adiposity. These findings highlight the impact of body composition on muscular EF and underscore the importance of weight management in mitigating musculoskeletal degeneration and LBP risk.

Female participants had notably higher total EF scores (28.6±9.9) compared to their male counterparts (22.1±11.2), with independent t-tests giving good statistical significance (p=0.025; t=2.28) as depicted in Table 5. This finding implies that women may be more prone to EF accumulation in the paraspinal muscles, which could influence the severity and management of LBP differently between sexes.

**Table 5:** Comparison of total EF scores by sex N: number of participants; EF: epimuscular fat; SD: standard deviation *: statistically significant

Sex	N	Mean total EF score±SD	P-value	t-value
Female	26	28.6±9.9	0.025^*^	2.28
Male	30	22.1±11.2		

EF scores demonstrated a significant variation across BMI categories. Participants in the normal weight range (BMI 18.5-24.9 kg/m²) had the lowest mean EF score (16±10.1), whereas those classified as obese participants (BMI ≥30 kg/m²) exhibited the highest mean EF score (32±12.5). Overweight participants (BMI 25-29.9 kg/m²) also showed elevated EF scores (25.4±15.2). Statistical analysis revealed a statistically significant difference in EF scores among the three BMI groups (p=0.006; F=17.36), indicating that EF increases with higher BMI, as depicted in Table 6.

**Table 6:** EF scores by BMI categories BMI: body mass index; N: number of participants; EF: epimuscular fat; SD: standard deviation *: statistically significant

BMI category	N	Mean EF score±SD	P-value	F-value
Normal	15	16±10.1	0.006^*^	17.36
Overweight	25	25.4±15.2		
Obese	16	32±12.5		

EF scores were evaluated in relation to LBP severity, categorized using the ODI. Participants with minimal pain had the lowest mean EF score (19.5±6.2), while those with moderate and severe pain exhibited progressively higher scores (26.4±11.4 and 31.5±10.5, respectively). The highest EF accumulation was noted in the crippling pain group (40±5.0). Statistical analysis revealed a significant difference in EF score distribution across the LBP severity categories (p=0.0036; F=5.23), indicating a clear trend of increasing EF with worsening pain severity. These findings suggest a strong association between EF accumulation and functional impairment due to chronic LBP, as depicted in Table 7.

**Table 7:** EF scores by LBP severity categories LBP: low back pain; ODI: Oswestry Disability Index; N: number of participants; SD: standard deviation *: statistically significant

LBP severity based on ODI percentage	N	Mean total EF score±SD	P-value	F-value
Minimal pain (0-20%)	11	19.5±6.2	0.0036^*^	5.23
Moderate pain (21-40%)	28	26.4±11.4		
Severe pain (41-60%)	15	31.5±10.5		
Crippling pain (61-80%)	2	40±5.0		
Bedbound pain (81-100%)	-	-		

A multiple linear regression analysis identified age, BMI, sex, and LBP severity as significant independent predictors of total EF scores in the paraspinal muscles. Age had a β coefficient of 0.35 (p=0.004); BMI had a β coefficient of 0.40 (p=0.001); sex (coded as female=1) had a β coefficient of 0.25 (p=0.035); and LBP severity showed a significant positive association with EF scores, with a β coefficient of 0.40 (p=0.010). Collectively, these variables explained 44% of the variance in EF scores (R²=0.44) as depicted in Table 8. These findings highlight the combined influence of demographic (age, BMI, sex) and clinical (LBP severity) factors on paraspinal muscle EF, emphasizing the need to consider both physiological and symptomatic components in evaluating chronic LBP.

**Table 8:** Multiple linear regression analysis for predictors of epimuscular fat scores BMI: body mass index; LBP: low back pain; R²: coefficient of determination *: statistically significant

Predictor	β coefficient	Standard error	P-value
Age	0.35	0.12	0.004^*^
BMI	0.40	0.10	0.001^*^
Sex (female=1)	0.25	0.11	0.035^*^
LBP severity	0.40	0.15	0.010^*^
Coefficient of determination	Value		
R²	0.44		
Adjusted R²	0.41		

## Discussion

This study investigated the distribution and clinical significance of EF in patients with chronic nonspecific LBP using MRI-based qualitative scoring. Among the 56 participants, the mean age was 45.3±12.7 years, with more male participants (53.6%). Most participating individuals come under the overweight category with an average BMI. Participants reported an average LBP duration of 12.5±6.8 months, with a mean ODI score of 33.2±25%, suggesting moderate functional limitation. These findings are similar to previous studies by Rosenstein et al. and Vitale et al., which report a higher prevalence of LBP among middle-aged and overweight populations [7,8].

A cranio-caudal pattern of EF deposition was observed, with the lowest fat deposition at L1-L2 (2.1±0.8) and the highest at L4-L5 (7.2±1.4) and L5-S1 (6.8±1.2). This distinct trend aligns with the hypothesis that lower lumbar segments are more prone to degenerative changes and EF accumulation due to active biomechanically load-bearing. Similar observations have been reported by Rosenstein et al. and Vitale et al., who documented increased EF deposition at the L3-S1 levels in LBP cohorts [7,8].

Significant positive correlations were found between total EF scores and both age (r=0.45; p<0.001) and BMI (r=0.52; p<0.001) on statistical analysis, indicating that EF accumulates with age and higher body fat levels. These results support prior research findings by Vitale et al. stating that paraspinal EF deposition is related to age-related and obesity-related metabolic effects and the relationship between EF and body composition underscores the potential role of weight management in reducing muscular degeneration in chronic LBP [8].

Women had significantly higher total EF scores (28.6±9.9) in comparison to men (22.1±11.2), with a p-value of 0.025. This sex-based difference in EF distribution could be attributed to hormonal influences and inherent differences in fat metabolism in women. Similar findings by Rosenstein et al. showed that women have increased EF deposition in paraspinal musculature in their study [7].

In our study, the EF scores also varied significantly across BMI categories. Participants classified as obese had the highest EF score (32±12.5), followed by overweight (25.4±15.2) and normal-weight individuals (16±10.1), with a p-value of 0.006. This gradation reinforces the direct relationship between systemic adiposity and localized fat deposition in paraspinal muscles.

A significant association was observed between EF scores and the severity of LBP as measured by ODI. Mean EF scores increased across ODI categories, from 19.5±6.2 in participants with minimal pain to 40±5.0 in those with crippling pain (p=0.0036), suggesting that increased FI may reduce muscle efficiency and contribute to pain persistence. This contrasts with findings from the work of Rosenstein et al., which reported no obvious relationship between EF and LBP severity [7]. However, our results aligned with the work of Vitale et al., who also found EF to correlate with pain intensity [8].

In our study, multiple linear regression analysis further confirmed age (β=0.35; p=0.004), BMI (β=0.40; p=0.001), sex (β=0.25; p=0.035), and LBP severity (β=0.40; p=0.010) as significant independent predictors of total EF scores. This had accounted for 44% of the variance in EF (R²=0.44), with 41% of the variance remaining after correction for the number of predictors (adjusted R²=0.41), highlighting the multifactorial nature of EF accumulation in LBP, with both modifiable (BMI and LBP severity) and non-modifiable (age and sex) factors contributing.

However, certain drawbacks of the present study must be acknowledged. Although the sample size was adequate for statistical analysis, the study was confined to a single tertiary care center. Additionally, while major spinal pathologies were excluded on MRI, subtle degenerative changes, such as early disc desiccation and mild osteophyte formation, were not systematically excluded and may have influenced EF distribution and LBP severity in some participants. Hence, larger multicenter studies with more refined imaging-based stratification are warranted to validate and extend these findings.

In summary, this study supports the growing body of evidence that EF accumulation is regionally patterned and clinically significant in chronic LBP. The findings demonstrate a strong link between EF and demographic and symptomatic factors. These results suggest that EF may serve as a potential biomarker of musculoskeletal health and a modifiable target in the management of chronic LBP. Further longitudinal studies are needed to establish the causative role of EF and assess the impact of targeted interventions on reducing FI and improving patient outcomes.

## Conclusions

This study elucidates a robust association between EF accumulation and variables such as age, BMI, sex, and the clinical severity of chronic LBP. The predilection of EF for lower lumbar segments implies a region-specific biomechanical adaptation, potentially exacerbating spinal instability and nociceptive burden. These findings accentuate the clinical imperative for comprehensive paraspinal musculature evaluation and posit EF as a promising biomarker and interventional target. Strategically addressing EF deposition may facilitate muscular rehabilitation, reinforce spinal architecture, and mitigate pain. Future longitudinal investigations with broader cohorts are essential to substantiate these observations and delineate causality.
